# Supplementary material for: Effects of diets on risks of cancer and the mediating role of metabolites
Source: Nat Commun. 2024 Jul 13;15:5903. doi: 10.1038/s41467-024-50258-4 (PMC11246454; doi:10.1038/s41467-024-50258-4)
Supplement: Supplementary file 1 — Supplementary Information [file 41467_2024_50258_MOESM1_ESM.pdf]

Supplementary materials for

Effects of Diet on Risk of cancer and the Mediating role of Metabolites

Yi Fan[1,2†], Chanchan Hu[1†], Xiaoxu Xie[1†], Yanfeng Weng[1†], Chen Chen[1], Zhaokun Wang[2], Xueqiong He[2], Dongxia Jiang[2], Shaodan Huang[2,3*], Zhijian Hu[1*], Fengqiong Liu[1*]

[1] Department of Epidemiology and Health Statistics, Fujian Provincial Key Laboratory of Environment Factors and Cancer, School of Public Health, Fujian Medical University, Fuzhou 350122, China;

[2] Department of Occupational and Environmental Health Sciences, School of Public Health, Peking University, Beijing 100191, China;

[3] Key Laboratory of Epidemiology of Major Diseases (Peking University), Ministry of Education, School of Public Health, Peking University, Beijing 100191, China;

†These authors contributed equally to this work.

* Corresponding author.

Fengqiong Liu. E-mail address: lfq@fjmu.edu.cn

Zhijian Hu. E-mail address: huzhijian@fjmu.edu.cn

Shaodan Huang. E-mail address: shhuang@bjmu.edu.cn

Supplementary Figures

Figure S1. Associations between diet components of MEDAS and MIND and overall cancer risk (N=187,485).

Figure S2. Associations between MEDAS and MIND scores and overall cancer risk stratified by basic characteristics (N=187,485).

Figure S3. Associations between MEDAS and MIND scores and 22 specific types of cancer risk stratified by basic characteristics

Figure S4. Selection of metabolites in in training set (N=121,382).

Figure S5. Associations between the MEDAS and MIND scores, metabolites, and overall cancer risk (N=85,669).

Figure S6. Flowchart of the study

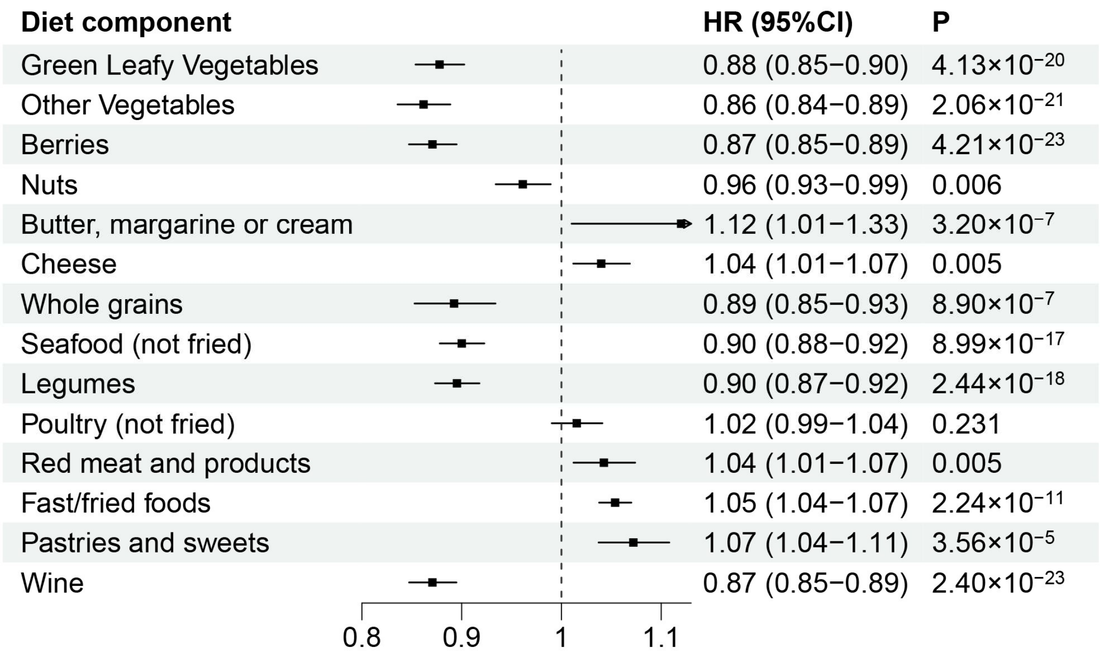

(a) Associations between diet components of MEDAS and overall cancer risk

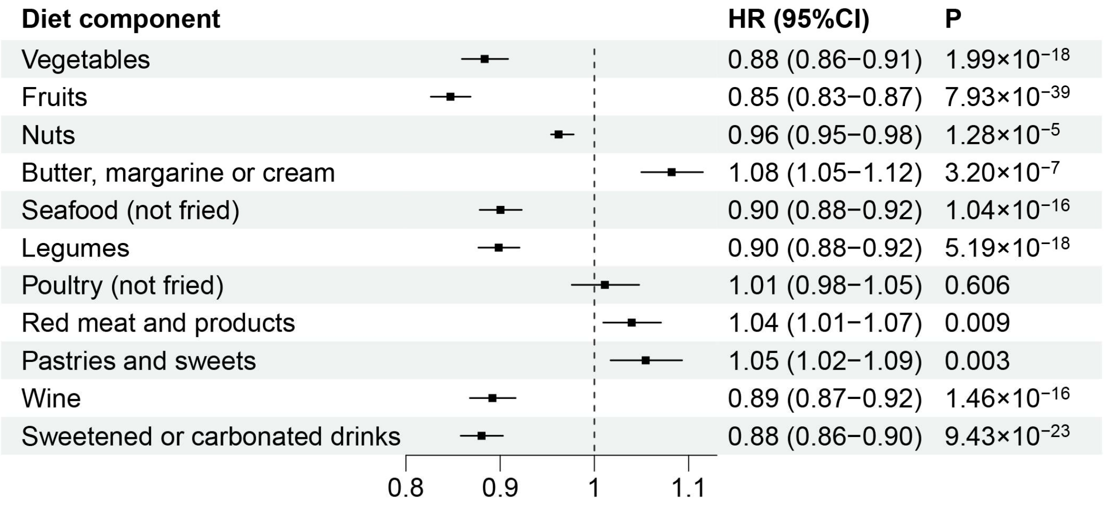

(b) Associations between diet components of MIND and overall cancer risk

Figure S1. Associations between diet components of MEDAS and MIND and overall cancer risk (N=187,485)

HR: hazard ratio; *CI*: confidence interval. The *HR* and 95%*CI* were estimated using Cox regression with adjustment for covariates in model 3 (two-sided Wald test). The gray dashed line represents the null (*HR* = 1). Each point shows the point estimate of *HR* from cox regression. Bars show 95%CI. Diet components of these two scores were treated as continuous in the model. Source data are provided with this publication as a Source Data file.

Subgroup	Diet Adherence	N	Incidence	P for interaction	HR (95%CI)
**Sex**					
Female	MEDAS score	101709	12437	0.480	0.900 (0.878−0.923)
Male	MEDAS score	85776	13954		0.884 (0.862−0.906)
Female	MIND score	101709	12437	0.018	0.918 (0.898−0.938)
Male	MIND score	85776	13954		0.880 (0.862−0.897)
**Completion of full−time education**					
No	MEDAS score	80992	10825	0.976	0.894 (0.870−0.919)
Yes	MEDAS score	106493	15566		0.895 (0.874−0.916)
No	MIND score	80992	10825	0.512	0.903 (0.882−0.924)
Yes	MIND score	106493	15566		0.896 (0.879−0.914)
**Family history of cancer**					
No	MEDAS score	138612	18667	0.451	0.898 (0.879−0.917)
Yes	MEDAS score	48873	7724		0.885 (0.856−0.914)
No	MIND score	138612	18667	0.573	0.901 (0.886−0.918)
Yes	MIND score	48873	7724		0.892 (0.868−0.917)
**Smoking status**					
No	MEDAS score	106872	13582	0.101	0.877 (0.856−0.899)
Yes	MEDAS score	80613	12809		0.902 (0.880−0.926)
No	MIND score	106872	13582	0.489	0.890 (0.871−0.908)
Yes	MIND score	80613	12809		0.899 (0.880−0.918)
**Alcohol drinking**					
No	MEDAS score	11695	1511	0.165	0.873 (0.806−0.946)
Yes	MEDAS score	175790	24880		0.897 (0.881−0.913)
No	MIND score	11695	1511	0.861	0.917 (0.859−0.979)
Yes	MIND score	175790	24880		0.899 (0.885−0.913)
**BMI**					
<25	MEDAS score	69907	9092	0.200	0.902 (0.875−0.929)
≥25	MEDAS score	117578	17299		0.885 (0.866−0.905)
<25	MIND score	69907	9092	0.131	0.908 (0.886−0.932)
≥25	MIND score	117578	17299		0.889 (0.873−0.906)

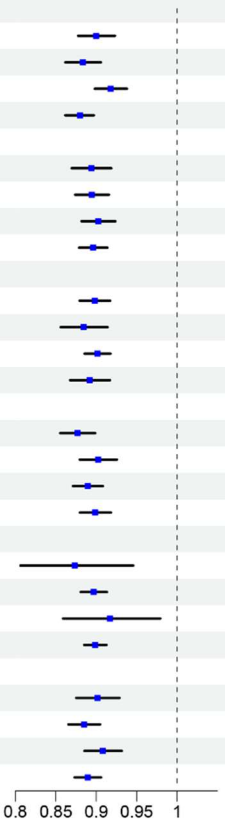

Figure S2. Associations between MEDAS and MIND scores and overall cancer risk stratified by basic characteristics (N=187,485)

BMI body mass index; *HR*: hazard ratio; *CI*: confidence interval; MEDAS: mediterranean diet adherence screener; MIND: Mediterranean-DASH Diet Intervention for Neurodegenerative Delay. The *HR* and 95%*CI* were estimated using Cox regression, adjusting for covariates in model 3, and including the cross-product term of MEDAS (or MIND) and the basic characteristics in multivariable regressions (two-sided Wald test). The gray dashed line represents the null ($HR = 1$). Each blue point shows the point estimate of *HR* from cox regression. Bars show 95%*CI*. Source data are provided with this publication as a Source Data file

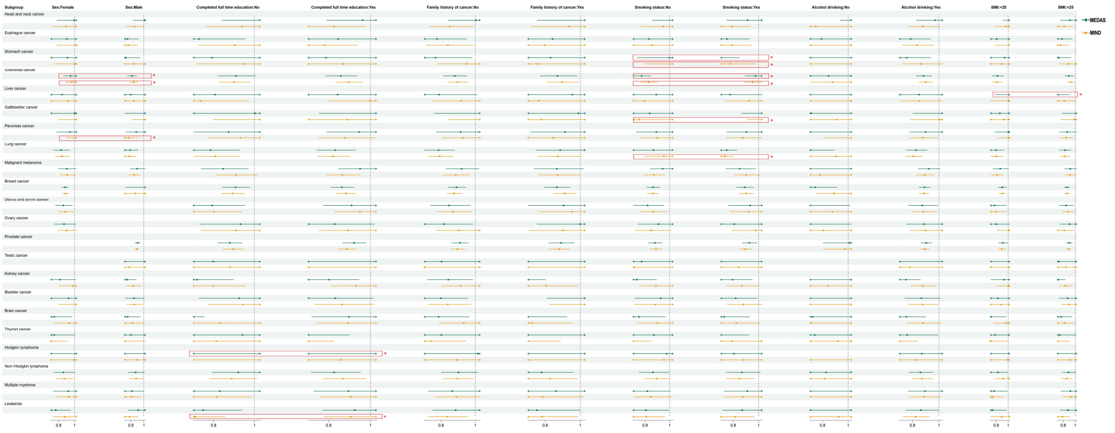

Figure S3. Associations between MEDAS and MIND scores and 22 specific types of cancer risk stratified by basic characteristics

BMI: body mass index; *HR*: hazard ratio; *CI*: confidence interval; MEDAS: mediterranean diet adherence screener; MIND: Mediterranean-DASH Diet Intervention for Neurodegenerative Delay. The *HR* and 95%*CI* were estimated using Cox regression, adjusting for covariates in model 3, and including the cross-product term of MEDAS (or MIND) and the basic characteristics in multivariable regressions (two-sided Wald test). The green color indicates MEDAS score, while the orange color indicates MIND score. The green blue represents MEDAS score, and the orange blue represents MIND score. The red box represents statistically significant interactions between MEDAS (or MIND) and basic characteristics and the details results were shown in Table S9. The sample size of study populations for each specific types of cancer risk are as follows: $N_{head\ and\ neck\ cancer}$=161440; $N_{esophagus\ cancer}$=161401; $N_{stomach\ cancer}$=; $N_{colorectal\ cancer}$=162960; $N_{liver\ cancer}$=161286; $N_{gallbaldder\ cancer}$=161196; $N_{pancreas\ cancer}$=161529; $N_{lung\ cancer}$=161094; $N_{malignant\ melanoma}$=162154; $N_{breast\ cancer}$=161094; $N_{uterus\ and\ cervix\ cancer}$=161417; $N_{ovary\ cancer}$=161424; $N_{prostate\ cancer}$=165267; $N_{testis\ cancer}$=161137; $N_{kidney\ cancer}$=161596; $N_{bladder\ cancer}$=161466; $N_{brain\ cancer}$=161382; $N_{thyroid\ cancer}$=161230; $N_{hodgkin\ lymphoma}$=161135; $N_{non\text{-}Hodgkin\ lymphoma}$=161879; $N_{multiple\ myeloma}$=161457; $N_{leukemia}$=161513. Source data are provided with this publication as a Source Data file.

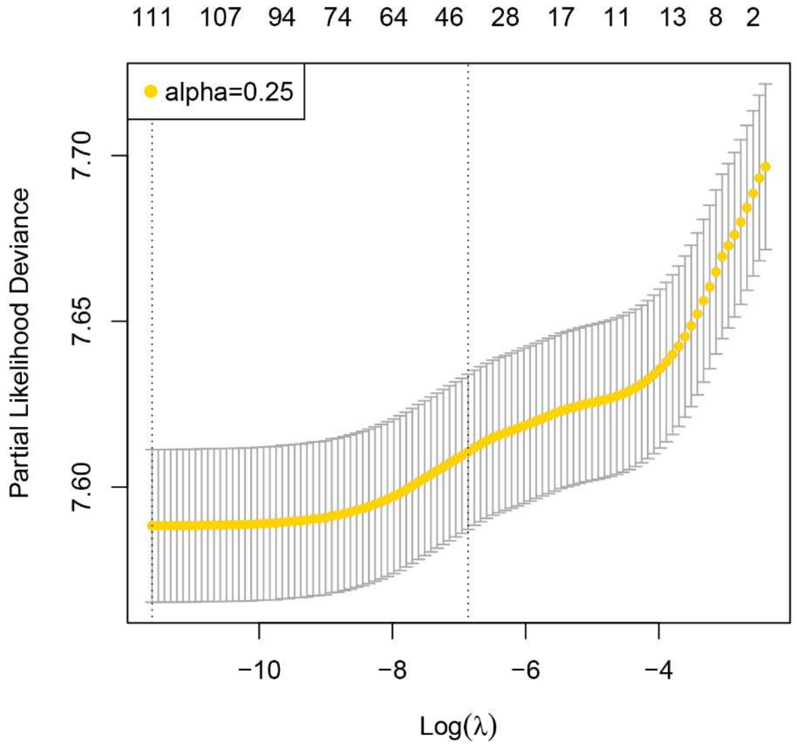

(a) Test α of 0.25 in ENM

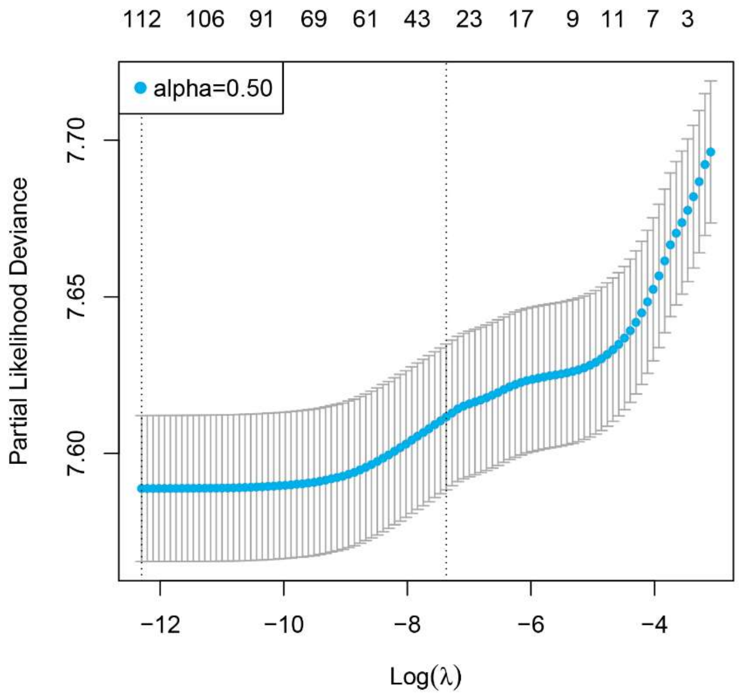

(b) Test α of 0.50 in ENM

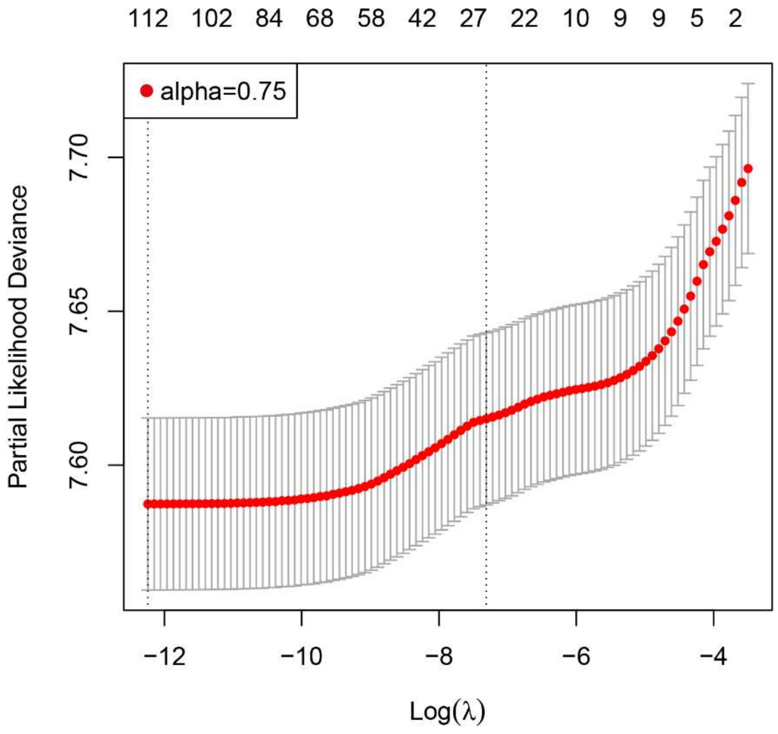

(c) Test α of 0.75 in ENM

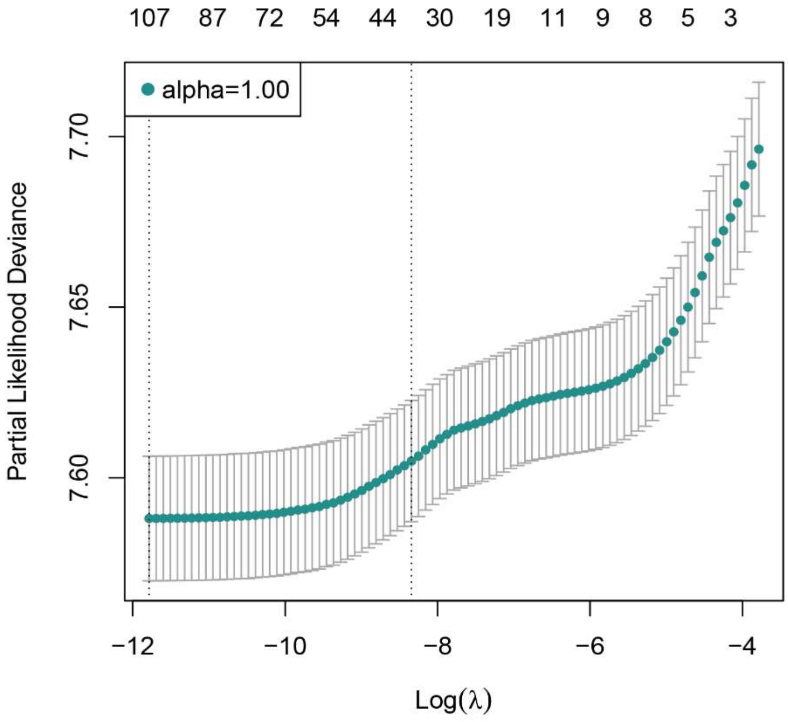

(d) Test α of 1.00 in ENM

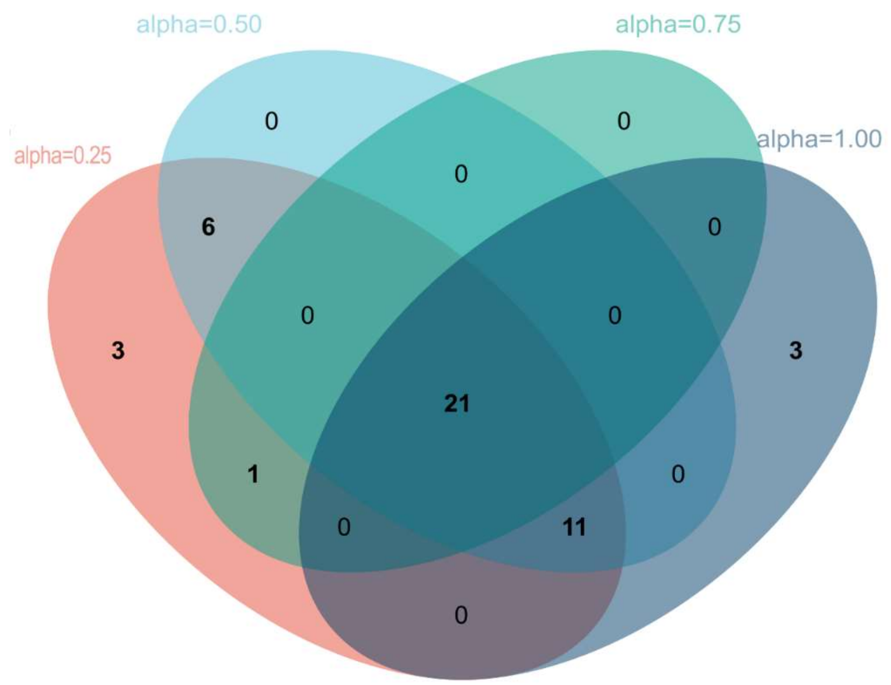

(e) Shared metabolites of α of 0.5, 0.75, and 1 in ENM

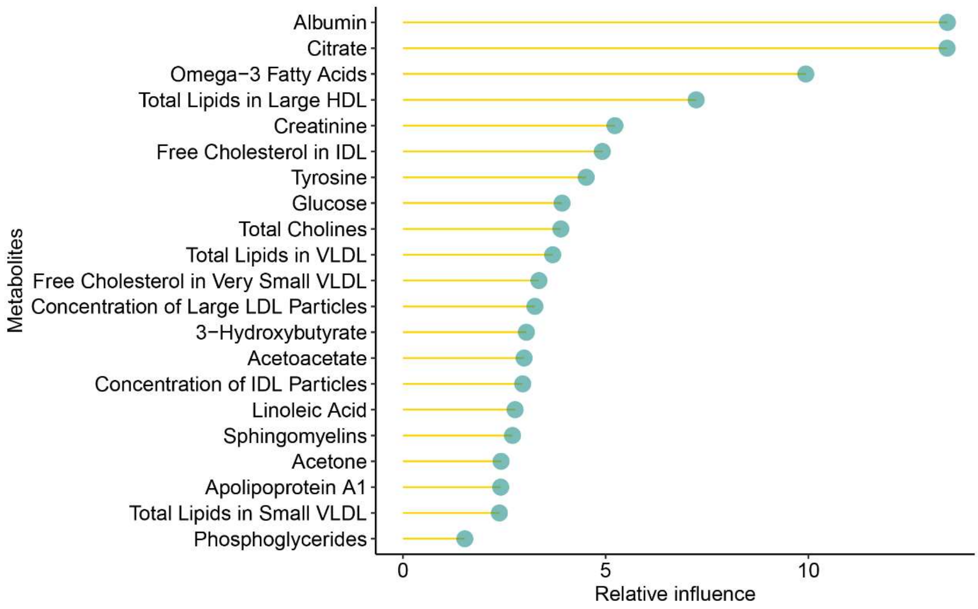

(f) The top 10 shared metabolites selected by gradient boosting trees

Figure S4. Selection of metabolites in in training set (N=121,382).

ENM: elastic net model. (a-d) 10-fold cross-validation ENM Cox regression models with α value of 0.25, 0.50, 0.75, and 1.00 were used to select metabolites. Metabolites with coefficients not equal to zero were selected. (e) Venn plot was used to visually display the shared metabolites with α values of 0.5, 0.75, and 1 in ENM (f) The Y-axis represents the shared metabolites with α values of 0.5, 0.75, and 1 in ENM, while the

X-axis represents the relative influence of 10-fold cross-validation gradient boosting trees using the Cox regression model as the underlying model. Source data are provided with this publication as a Source Data file.

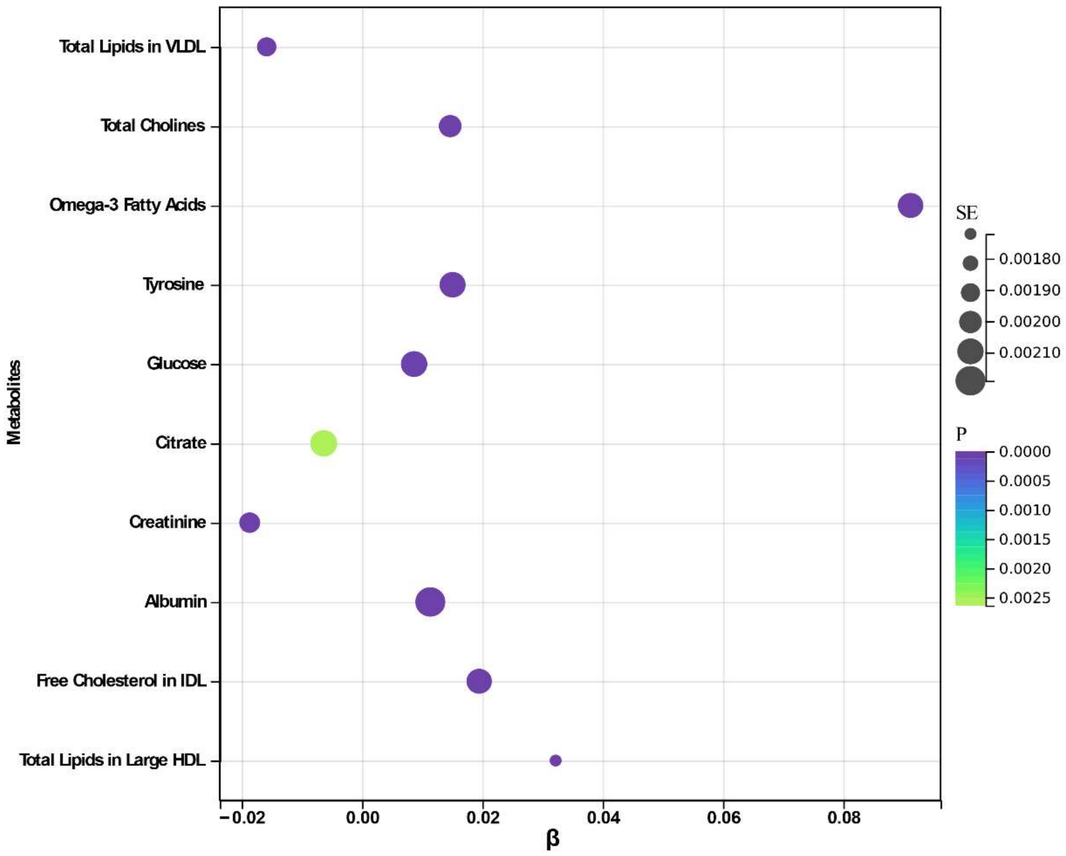

(a) Association between metabolites and MEDAS score

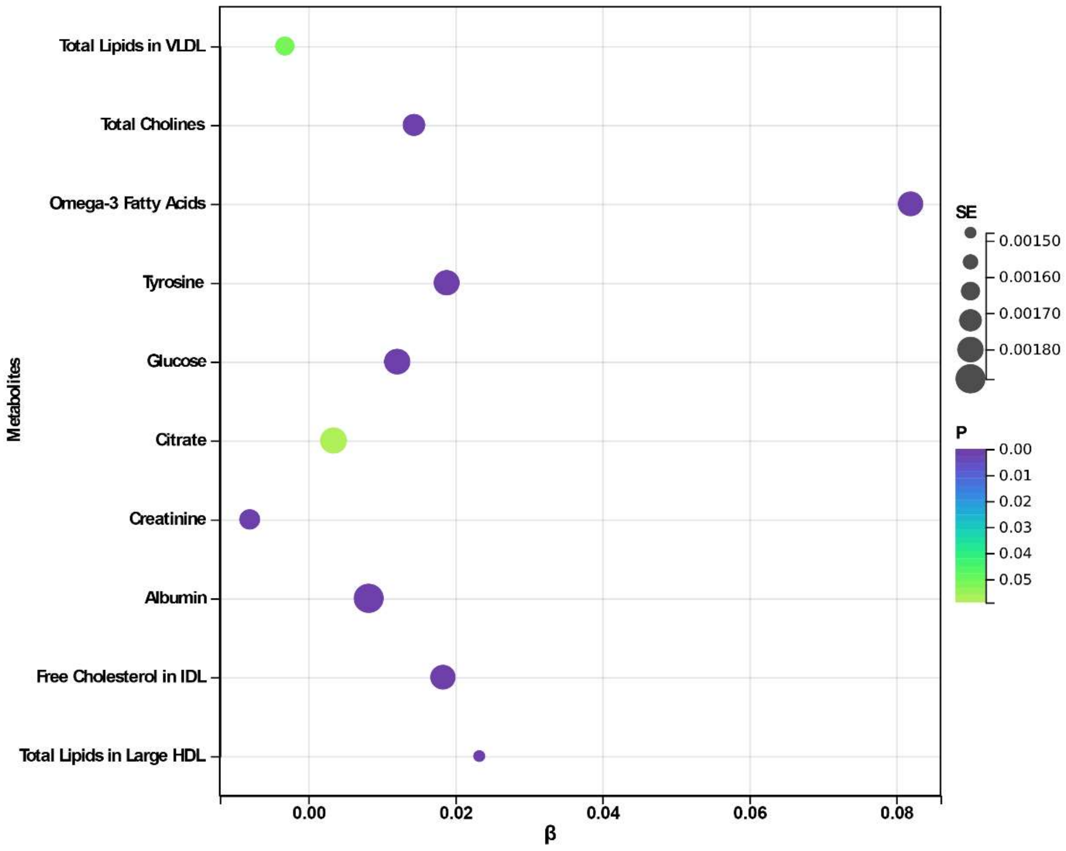

(b) Association between metabolites and MIND score

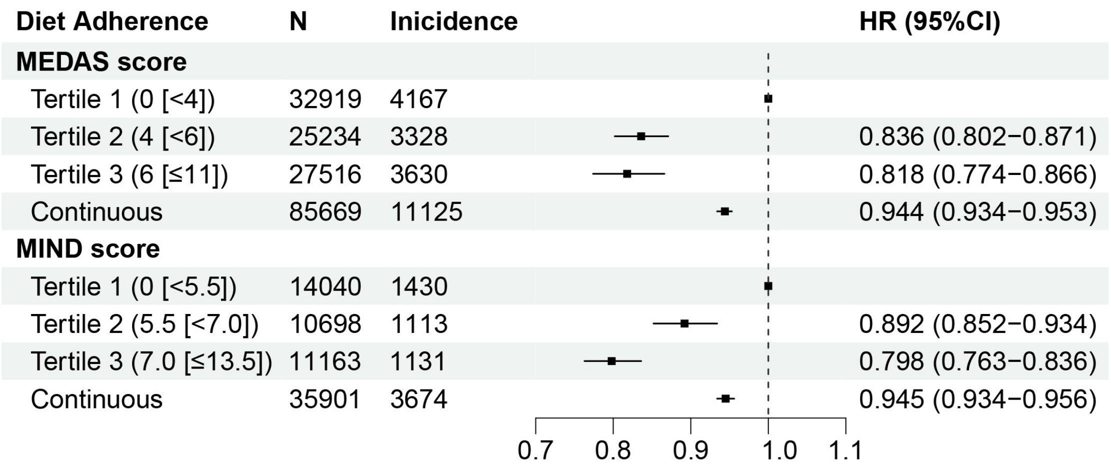

(c) Associations between MEDAS and MIND score and overall cancer risk

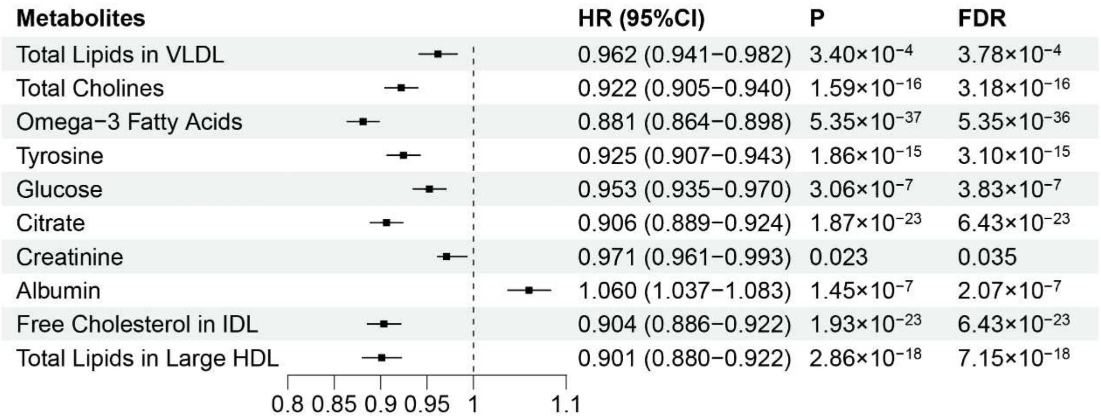

(d) Associations between metabolites and overall cancer risk

Figure S5. Associations between the MEDAS and MIND scores, metabolites, and overall cancer risk (N=85,669)

(a-b) *SE*: standard error. Multivariable linear regression was used to obtain the β coefficient and *SE* of the metabolites (two-sided Wald test). The Y-axis represents the metabolites, while the X-axis represents β coefficient of these metabolites. The point size represents the *SE* of these metabolites, the point color represents the *p*-value. (c) HR: hazard ratio; CI: confidence interval; MEDAS: mediterranean diet adherence screener; MIND: Mediterranean-DASH Diet Intervention for Neurodegenerative Delay. The *HR* and 95%*CI* were estimated using Cox regression with adjustment for covariates in model 3 (two-sided Wald test). Each point shows the point estimate of *HR* from cox regression. Bars show 95%CI. (d) *FDR*: false discovery rate. The *HR* and 95%*CI* of metabolites (treated as continuous in model) were estimated using Cox regression with

adjustment for covariates in model 3 . Each point shows the point estimate of *HR* from cox regression. Bars show 95%CI. *FDR* is an indicator used to adjust *p*-value and to control for false detective rate in multiple hypothesis testing. Associations were considered significant when *FDR*<0.05 (two-sided Wald test). Source data are provided with this publication as a Source Data file.

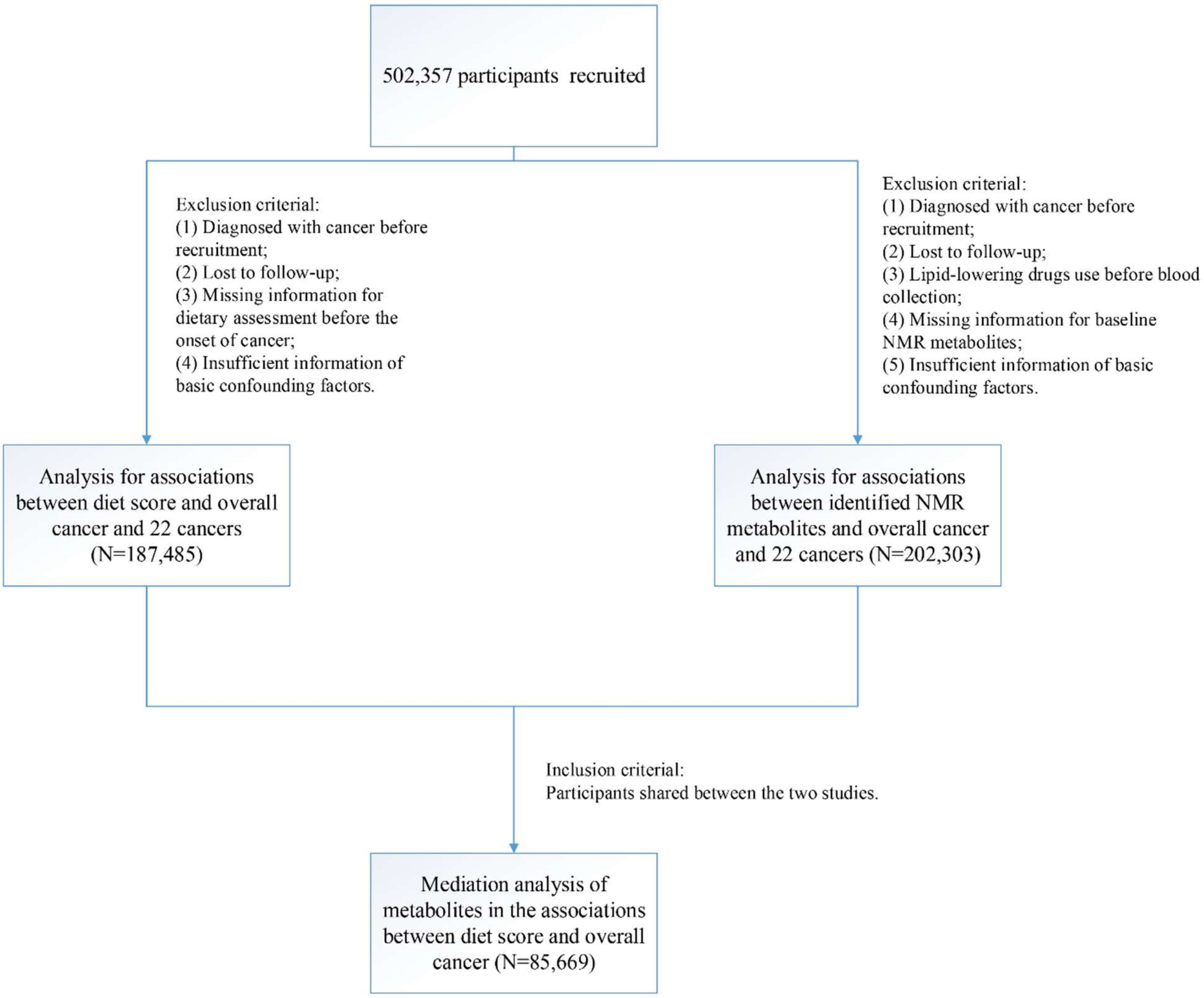

Figure S6. Flowchart of the study
