# Supplementary material for: Effects of diets on risks of cancer and the mediating role of metabolites
Source: Nat Commun. 2024 Jul 13;15:5903. doi: 10.1038/s41467-024-50258-4 (PMC11246454; doi:10.1038/s41467-024-50258-4)
Supplement: Supplementary file 4 — Reporting Summary [file 41467_2024_50258_MOESM4_ESM.pdf]

nature portfolio

Corresponding author(s): Fengqiong Liu, Hu zhijian, Huang Shaodan

Last updated by author(s): Apr 12, 2024

Reporting Summary

Nature Portfolio wishes to improve the reproducibility of the work that we publish. This form provides structure for consistency and transparency in reporting. For further information on Nature Portfolio policies, see our Editorial Policies and the Editorial Policy Checklist.

Statistics

For all statistical analyses, confirm that the following items are present in the figure legend, table legend, main text, or Methods section.

n/a	Confirmed	
☐	☒	The exact sample size (*n*) for each experimental group/condition, given as a discrete number and unit of measurement
☐	☒	A statement on whether measurements were taken from distinct samples or whether the same sample was measured repeatedly
☐	☒	The statistical test(s) used AND whether they are one- or two-sided<br>*Only common tests should be described solely by name; describe more complex techniques in the Methods section.*
☐	☒	A description of all covariates tested
☐	☒	A description of any assumptions or corrections, such as tests of normality and adjustment for multiple comparisons
☐	☒	A full description of the statistical parameters including central tendency (e.g. means) or other basic estimates (e.g. regression coefficient) AND variation (e.g. standard deviation) or associated estimates of uncertainty (e.g. confidence intervals)
☐	☒	For null hypothesis testing, the test statistic (e.g. *F*, *t*, *r*) with confidence intervals, effect sizes, degrees of freedom and *P* value noted<br>*Give P values as exact values whenever suitable.*
☒	☐	For Bayesian analysis, information on the choice of priors and Markov chain Monte Carlo settings
☒	☐	For hierarchical and complex designs, identification of the appropriate level for tests and full reporting of outcomes
☐	☒	Estimates of effect sizes (e.g. Cohen's *d*, Pearson's *r*), indicating how they were calculated

Our web collection on statistics for biologists contains articles on many of the points above.

Software and code

Policy information about availability of computer code

Data collection	All data was previously collected and is described in detail elsewhere (UK Biobank). The dataset analysed during this study is available in the UK Biobank (https://www.ukbiobank.ac.uk/) under application number 675116. Raw data from the UK Biobank cannot be shared per our Material Transfer Agreement; Access can be obtained by data application for the UK Biobank platform.
Data analysis	R version 4.1.1, and Python v3.11;<br>R packages: data.table version 1.15.0, survival version 3.5.8, tidyverse version 2.0.0, mice version 3.16.0, tableone version 0.13.2, glmnet version 4.1.8, caret version 6.0.94, mma version 10.7.1, grid version 4.3.2, forestploter version 1.1.1, ggsci version 3.0.0, ggplot2 version 3.5.0, gbm version 2.1.9, viridis version 0.6.5;<br>Python packages: pandas version 2.1.4, tqdm version 4.66.1, datetime version 5.4;<br>Code: https://doi.org/10.5281/zenodo.10953469.

For manuscripts utilizing custom algorithms or software that are central to the research but not yet described in published literature, software must be made available to editors and reviewers. We strongly encourage code deposition in a community repository (e.g. GitHub). See the Nature Portfolio guidelines for submitting code & software for further information.

Data

Policy information about [availability of data](#)

All manuscripts must include a [data availability statement](#). This statement should provide the following information, where applicable:

- Accession codes, unique identifiers, or web links for publicly available datasets
- A description of any restrictions on data availability
- For clinical datasets or third party data, please ensure that the statement adheres to our [policy](#)

The dataset analysed during this study is available in the UK Biobank (https://www.ukbiobank.ac.uk/) under application number 675116. Raw data from the UK Biobank cannot be shared per our Material Transfer Agreement; access can be obtained by application through the UK Biobank platform. Source data supporting all our findings (Figures 1–4 and Figure S1–S5) are provided with this publication as a Source Data file.

Research involving human participants, their data, or biological material

Policy information about studies with human participants or human data. See also policy information about sex, gender (identity/presentation), and sexual orientation and race, ethnicity and racism.

Reporting on sex and gender	Sex and gender were not considered in the study design, self-reported sex information was used in the study.
Reporting on race, ethnicity, or other socially relevant groupings	Race, ethnicity, or other socially relevant groupings were not considered in the study design.
Population characteristics	1. Aged from 37 to 73 years old, averaged 57 years old.<br>2. With 54% female and 46% males<br>3. With a median follow up time of 13.2 years<br>4. 26% of participants with cancer family history<br>5. 57% of participants with completed full time education
Recruitment	Our study population is based on the UK Biobank, which recruited over 500,000 participants from 22 assessment centers from England, Wales and Scotland, thus it is less likely to have selection bias during recruitment.
Ethics oversight	The study was approved by the NHS National Research Ethics Service (Ref:11/NW/0382)

Note that full information on the approval of the study protocol must also be provided in the manuscript.

Field-specific reporting

Please select the one below that is the best fit for your research. If you are not sure, read the appropriate sections before making your selection.

☒ Life sciences ☐ Behavioural & social sciences ☐ Ecological, evolutionary & environmental sciences

For a reference copy of the document with all sections, see nature.com/documents/nr-reporting-summary-flat.pdf

Life sciences study design

All studies must disclose on these points even when the disclosure is negative.

Sample size	First, we investigated the associations between diet scores and risk of overall, and 22 specific cancers. After excluding participants who had a pre-existing cancer diagnosis, those lost to follow up, individuals with missing dietary assessment information before cancer onset, or those with missing information for more than 80% covariates, we were left with a study population of 187,485 participants. Second, we identified metabolites associated with overall cancer, from the original pool of 168 metabolites. For this analysis we excluded participants who had a pre-existing cancer diagnosis, were lost to follow up, used lipid-lowering drugs before blood collection, and those with missing data on baseline metabolites. Additionally, we excluded participants lacking information on more than 80% of the basic confounding factors. The total number of participants included was 202,303. Finally, we explored mediating roles of the identified metabolites in how diet affects overall cancer risk. This final analysis involved 85,669 participants, constituting a shared subset between the two aforementioned studies, who had both diet assessment and metabolites data. The flowchart of our study design is shown in Figure S5. It should be pointed out that, though the numbers of study populations varied for each of the analyses due to the differing missing variables among participants, the statistic characteristics of the populations in the 3 analyses were very similar, as can be seen from Table 1 and Tables S1-S4.
Data exclusions	1. Participants with missing data of diet assessment.<br>2. Participants only had diet assessment data after cancer onset.<br>3. Participants with pre-existing cancer<br>4. Participants who lost follow-up<br>5. Particpants with insufficient information for covariate analysis<br>6. Participant with missing information for NMR metabolites
Replication	We performed 10 sensentivity analysis with different definition to test the robustness of our finds, and main findings could be replicated . And all analysis were cross validated by two independent data analyst.

Randomization	This is no relevant since this is a prospective design and we focused on correlation analysis instead of grouping difference.
Blinding	This research adopts an open design.

Reporting for specific materials, systems and methods

We require information from authors about some types of materials, experimental systems and methods used in many studies. Here, indicate whether each material, system or method listed is relevant to your study. If you are not sure if a list item applies to your research, read the appropriate section before selecting a response.

Materials & experimental systems

n/a	Involved in the study	
☒	☐	Antibodies
☒	☐	Eukaryotic cell lines
☒	☐	Palaeontology and archaeology
☒	☐	Animals and other organisms
☒	☐	Clinical data
☒	☐	Dual use research of concern
☒	☐	Plants

Methods

n/a	Involved in the study	
☒	☐	ChIP-seq
☒	☐	Flow cytometry
☒	☐	MRI-based neuroimaging

Plants

Seed stocks	*Report on the source of all seed stocks or other plant material used. If applicable, state the seed stock centre and catalogue number. If plant specimens were collected from the field, describe the collection location, date and sampling procedures.*
Novel plant genotypes	*Describe the methods by which all novel plant genotypes were produced. This includes those generated by transgenic approaches, gene editing, chemical/radiation-based mutagenesis and hybridization. For transgenic lines, describe the transformation method, the number of independent lines analyzed and the generation upon which experiments were performed. For gene-edited lines, describe the editor used, the endogenous sequence targeted for editing, the targeting guide RNA sequence (if applicable) and how the editor was applied.*
Authentication	*Describe any authentication procedures for each seed stock used or novel genotype generated. Describe any experiments used to assess the effect of a mutation and, where applicable, how potential secondary effects (e.g. second site T-DNA insertions, mosiacism, off-target gene editing) were examined.*
